# Health-related quality of life among colorectal cancer patients in Malaysia: a study protocol

**DOI:** 10.1186/1471-2407-12-384

**Published:** 2012-09-03

**Authors:** Bello Arkilla Magaji, Foong Ming Moy, April Camilla Roslani, Ismail Sagap, Jasiah Zakaria, Jane M Blazeby, Chee Wei Law

**Affiliations:** 1 Julius Centre University of Malaya, Department of Social & Preventive Medicine, Faculty of Medicine, University of Malaya, 50603, Kuala Lumpur, Malaysia; 2 Department of Surgery, Faculty of Medicine, University of Malaya, 50603, Kuala Lumpur, Malaysia; 3 UMCRI (University of Malaya Cancer Research Institute), Kuala Lumpur, Malaysia; 4 Academic Unit of Surgical Research, School of Social and Community Medicine, University of Bristol, Bristol, UK; 5 Division of Surgery, Head & Neck, University Hospitals Bristol NHS Foundation Trust, Bristol, UK; 6 Colorectal Surgery, UKM Medical Center, Universiti Kebangsaan Malaysia, Jalan Yaacob Latif, Bandar Tun Razak, 56000, Cheras, Kuala Lumpur, Malaysia; 7 Department of Surgery, Hospital Tuanku Jaafar, Jalan Rasah, 70300, Seremban, Negeri Sembilan, Malaysia

**Keywords:** Colorectal cancer, Health related quality of life, Malaysia

## Abstract

**Background:**

Colorectal cancer is a major public health problem in Malaysia. However, it is also one of the most treatable cancers, resulting in significant numbers of survivors. Therefore, the impact of surviving treatment for colorectal cancer on health related quality of life is important for the patients, clinicians and policy makers, and may differ in different cultures and populations. The aim of this study was to validate the Malaysian versions of the European Organization for Research and Treatment of Cancer quality of life instruments among colorectal cancers patients.

**Methods/design:**

This is a cross sectional multi centre study. Three hospitals were included, the University of Malaya Medical Centre, the Universiti Kebangsaan Malaysia Medical Centre and Hospital Tuanku Jaafar Seremban. Malaysian citizens and permanent residence were studied and demographic and clinical information obtained from hospital records. The European Organization for Research and Treatment of Cancer Quality of life Core 30, colorectal cancer CR29, and the colorectal cancer liver metastasis LMC 21 were used and an observer assessment of performance obtained with the Karnofsky Performance Scale. Questionnaires were translated into three most commonly spoken languages in Malaysia (Bahasa Malaysia, Chinese and Tamil), then administered, scored and analyzed following the developers’ guidelines. Ethical approval was obtained from the participating centres. Tests of reliability and validity were performed to examine the validity of these instruments.

**Conclusion:**

The result of pilot testing shows that the use of the Malaysian versions of EORTC QLQ C30, CR29 instruments is feasible in our sample of colorectal cancer patients. Instructions for completion as well as questions were well understood except the questions on the overall quality of life, overall health status and sexual activity. Thus we anticipate obtaining good psychometric properties for the instruments at the end of the study.

## Background

Colorectal cancer is a major public health problem in Malaysia. Colon cancer ranked third among cancers reported in males and females, accounting for 7.8% and 5.6% in males and females respectively. Cancer of the rectum ranked fifth and eighth among cancers reported in males and females respectively. This disease accounted for 6.4% and 3.4% in males and females respectively. When taken together, colorectal cancers would account for 14.2% of male cancers making it the commonest cancer among men and 10.1% of female cancers the third most common cancer among women [1].

Conventional outcome measures focus on disease-centric criteria, such as complications or survival. However, the impact of treatments on patient wellbeing is rarely assessed, even though it may be just as significant if not more so. Studies on Health -related quality of life (HRQoL) is complementary to the traditional medical assessments rather than a standalone way of assessing well-being [2,3].

In assessing HRQoL it is important to use a valid measurement tool [4,5]. There are valid instruments that are in use presently, for example the Functional Assessment of Cancer Therapy consists of a core instrument (FACT-G) and its various sub-scales such as the FACT-C for colorectal cancer [6]. Another instrument is the European Organization for Research and Treatment of Cancer (EORTC), Quality of Life (QLQ) core questionnaire EORTC QLQ-C30 and several other modules. With respect to colorectal cancer, EORTC developed a colorectal cancer specific module EORTC QLQ-CR 29 and colorectal cancer liver metastasis module EORTC QLQ-LMC 21. However, in many cases, the validity and cultural context underlying developing such instruments are those of the original language and cultural setting [5,7]. This limits the direct application of such instruments. This brings about the need for translation and validation of these tools to suit the local needs and languages barriers. So far these questionnaires have been translated and validated in Europe and other parts of the world. However, such instruments are yet to be validated in Malaysia.

Therefore, this study aimed to validate the translated Malaysian versions of the EORTC QLQ-C30, EORTC QLQ-CR29 (version 2.1) and colorectal cancer liver metastasis EORTC QLQ-LMC21 instruments for health related quality of life measurement among colorectal cancer patients.

### Objectives

Our objective is to validate a Bahasa Malaysia, Chinese (Malaysia) and Tamil (Malaysia) versions of colorectal cancer disease EORTC QLQ-CR29 (version 2.1) and colorectal cancer liver metastasis EORTC QLQ-LMC21 instruments for health related quality of life measurement.

## Methods

### Setting

This is a multi-center study, involving three hospitals in Malaysia. The selected hospitals are University of Malaya Medical Centre (UMMC), Universiti Kebangsaan Malaysia Medical Centre (UKMMC) and Hospital Tuanku Jaafar, Seremban (HTJS).

### Study population (inclusion or exclusion)

The study includes Malaysian citizens or permanent residents with histologically confirmed colon or rectal cancers. They should be receiving or planned to receive at least one form of treatment at the medical centers. Excluded patients are those aged less than 18 years, those with incomplete diagnosis and those with language problem or inability to understand any of the three languages of the instruments.

### Ethical issues

This study was approved by the ethics committees of the UMMC (MEC Ref.No:770.2), UKMMC (Project code: FF-274-2011) and the Ministry of Health Malaysia for using Hospital Tuanku Jaafar Seremban (NMRR-11-348-9245). The project was also under the guidance of the EORTC QOL office. We obtained written, informed consent from each participant as recommended by the ethics of medical research.

### Study variables

A data extraction form was developed for the purpose of gathering the relevant demographic and clinical data from the hospital records. Variables collected were: patient’s identification number, age, sex, race, marital status, nationality, educational status, employment status and cohabitation. Information on the index cancer was: site of primary cancer (according to the IARC and UICC cancer classification manual 8th edition), tumour stage (Dukes), and histopathological differentiation. Treatment planned or received such as; surgery, presence or absence of stoma, chemotherapy and radiotherapy.

### Research tools

The instruments selected for this study include a ‘core’ instrument EORTC QLQ-C30 (version 3.0) and two other ‘modules’, for colorectal cancer EORTC QLQ-CR29 and colorectal cancer liver metastasis (LMC 21) respectively. The choice of these instruments was guided by the availability, established psychometric properties, and non-superiority of other instruments [7,8]. The QLQ-C30 is one of the most widely used instruments in cancer clinical research [8]. EORTC QLQ-CR29 is the newest version of the colorectal cancer specific QoL questionnaire recently validated in Europe [9]. The QLQ-LMC21 is the only liver metastasis specific instrument in use at present [10]. A Karnofsky Performance Scale was used by the clinicians to rate the well-being of the patients [11].

### Sample size estimation

1. **Pilot study:** according to the EORTC QOL group, each translated item of the questionnaire should be pilot-tested on 10 to 15 subjects before being field-tested on a larger sample [12]. So for each of the two set of questionnaires: EORTC QLQ-CR29 and LMC21, we have included 30 subjects, ten each for Bahasa Malaysia, Chinese and Tamil respectively

1. 2. **Validation:** For a multivariate analysis technique to gain reliable estimates, the number of subjects’ observations should be 10 times the number of variables in the model [13]. Therefore, the sample size was estimated based on this recommendation as follows;

a) **EORTC QLQ-CR29:** There are 29 items in the CR29. Thus the minimum number of subject required is 290. In our study, we use 300 subjects to account for possible attrition.

b) **EORTC QLQ-LMC 21:** This questionnaire contains 21 items; therefore, we need a minimum of 210 subjects for its validation.

### Procedure

#### Research assistants

Three research assistants aided in data collection. Each assistant could read and speak a minimum of two languages from the three languages used in this study.

### Patient’s identification

Prospective patients were identified using the eligibility criteria above. Eligible subjects received an invitation letter and/or telephone calls at least two weeks before their next visit to the clinic.

### Baseline data and Karnofsky Performance Scale

Baseline data for all prospective patients were obtained from the medical records using a data extraction form mentioned above. Missing data was obtained from the patients during the interview. The clinician completed the Karnofsky Performance scale during patient’s visit on the same day as the day of the interview.

### Questionnaire administration

Personal characteristics such as sexual behavior and family life are better assessed through a self-administered method. It has the advantages of being reliable, preferred, in HRQoL studies and also cheap to undertake [5]. The data collection method was self-administered and interviewer delivered the research administrators (research assistants and the researcher) presented the instruments, answered questions from respondents and were present throughout the sessions. This gave further motivation to the patients and encouraged them to answer every item. The reliability and quality in the answer were therefore ensured.

### Pilot study

In preparation for the pilot study, we translated the chosen tools into three main Malaysian languages based on the recommendations contained in the EORTC QoL group translation procedure [13].

The aim of pilot testing is to identify any potential problems in the translation of the instruments. Attention was on the following six areas; acceptability (face validity), clarity of the introduction and instructions, completeness, linguistic clarity, spontaneity of response and practicality of using the instruments. Interviews were conducted and it covered the following areas: difficulty in answering the questions, confusion, difficult words, upsetting nature, and an open comment on how the patient would ask a similar question if given opportunity. Responses were recorded and reported.

### Subject grouping and assessment plan

Eight groups of patients were examined. These were adopted and modified from the EORTC QLQ-CR29 validation study in Europe [9].

a) **Group 1**: patients with colon cancers, had surgery with no stoma, not receiving any form of chemotherapy. This group was being assessed once and the questionnaires were completed within 12 months of surgery.

b) **Group 2**: patients with colon cancers, had surgery with no stoma, receiving any form of chemotherapy. This group was being assessed once and the questionnaires were completed within 12 months of surgery but within two weeks of receiving chemotherapy.

c) **Group 3**: Rectal cancer patients with preoperative radiotherapy, the questionnaires were completed within two weeks of the radiotherapy.

d) **Group 4**: Patients with a permanent stoma; irrespective of the cancer site. This group was being assessed within five years post-surgery.

e) **Group 5:** Temporary stoma patients; irrespective of the cancer site. This group was being assessed twice, within one month after surgery with stoma, and within one to three months after closure of the stoma.

f) **Group 6**: Palliative care group: these were patients who were being treated with palliative intention. They were being assessed twice, first within two weeks of receiving chemotherapy or radiotherapy and second assessment was performed three months after the first assessment.

g) **Group 7:** Test-retest group; these patients were selected randomly from the six groups above and they were requested to complete the questionnaires within 7–14 days after the first assessment.

h) **Group 8**: Liver metastasis group; these patients were required to complete the questionnaires once.

### Statistical analysis

### Data preparation

Questionnaires were checked and data entered into a database in Microsoft Excel and later transferred to SPSS version 20.0 for Windows for analysis. Data coded based on the guidelines as contained in the EORTC scoring manual [14]. Two-sided tests were used, and p-values of ≤ 0.05 were considered statistically significant.

### Descriptive statistics

Descriptive statistical analysis was performed for all variables. Continuous variables were reported using means and standard deviations or median and inter-quartile range. For dichotomous variables, absolute numbers and percentages were presented**.** Completion rate and time taken to complete the questionnaires were assessed [15]. Confirmatory factor analysis was performed.

### Psychometric properties

#### Multitrait scaling analysis

Multitrait scaling analysis was employed to examine item convergent validity. Each items scale’s Pearson’s product moment correlation should exceed 0.4 for convergent validity on all scales. Inter scale correlations were used to measure discreminant validity which was a measure of item own scale correlation in relation to other scales. It is hypothesized item own correlation should be higher than with the other scales.

### Internal consistency reliability

The internal consistency was assessed using the cronbach’s alpha coefficient. Coefficients of above 0.70 were considered acceptable for group comparisons.

### Reproducibility (test-retest reliability)

Intraclass Correlation Coefficient (ICC) was used to assess the test-retest reliability. A score of one indicates perfectly reliable, zero perfectly unreliable test.

### Group comparisons

Subjects were compared based on the treatment groups such as subjects receiving chemotherapy, versus surgery alone, patients with and without stoma and performance status (KPS score of ≤ 80% versus ≥ 81%). Non parametric test Wilcoxon rank sum test were used for the comparison of the groups.

### Responsiveness

Responsiveness was measured by comparing changes over time of the instruments in subgroup of patients undergoing palliative chemotherapy (Group 6) and after closure of a temporary stoma (Group 4).

### Instrument acquisition and pilot testing

This study was designed to validate three EORTC questionnaires on a sample of colorectal cancer patients in Malaysia. The questionnaires included were; EORTC QLQ-C30 (version 3.0), EORTC QLQ CR29 and EORTC QLQ LMC21.

Questionnaires were acquired from the relevant developers and EORTC Quality of life group. Translation was performed according to the group guidelines; pilot testing was conducted and reported below. The validation testing is ongoing.

### Pilot testing

Pilot testing was conducted from January 02, 2012 to January 31, 2012. Questionnaires were administered to 10 patients for Bahasa Malaysia and 10 patients for Chinese-Malaysia versions. Due to inadequate number of patients suitable for QLQ LMC21 questionnaire and the Tamil version of all three questionnaires, we decided to remove them from our study.

### Patients’ characteristics

Details of the Socio demographic and clinical characteristics of the patients were presented in Table 1.0 for Bahasa Malaysia and Chinese-Malaysia.

**Table 1:** Socio demographic and clinical characteristics of patients included in the pilot testing of the EORTC QLQ-CR29 (Bahasa-Malaysia and Chinese-Malaysia)

**Variable**	**Bahasa-Malaysia**	**Chinese-Malaysia**
	**N (%)**	**N (%)**
**Age (years)**		
Mean (SD)	58 ± 12	67 ± 8
**Gender**		
Male	5(50)	5(50)
Female	5(50)	5(50)
**Education status**		
Primary	4(40)	1(10)
Secondary	3(30)	2(20)
Tertiary	3(30)	7(70)
**Employment status**		
Full time	4(40)	9(90)
Retired	6(60)	1(10)
**Tumour site**		
Ascending colon	-	2(20)
Transverse colon	-	2(20)
Splenic flexure	-	1(10)
Sigmoid	3(30)	-
Recto sigmoid	1(10)	2(20)
Rectum	6(60)	3(30)
**Stoma**		
Yes	5(50)	5(50)
No	5(50)	5(50)
**Chemotherapy**		
Yes	7(70)	6(60)
No	3(30)	4(40)
**Radiotherapy**		
Yes	6(60)	4(40)
No	4(40)	6(60)
KPS*		
≤80	1(10)	5(50)
≥80	9(90)	5(50)

*Karnofsky Performance Scale.

### Bahasa-Malaysia version

Mean age was 58 ± 12 years, Male: Female ratio 1:1, 30% had attained tertiary education, 60% had rectal cancer followed by sigmoid 30% and recto sigmoid 10% respectively. 50% had stoma, 70% chemotherapy and 60% had radiotherapy. Karnofsky performance status was ≥80 in 90% of patients.

### Chinese-Malaysia version

Mean age was 67 ± 8 years, Male: Female ratio 1:1, 70% had attained tertiary education, rectal cancer 30%, recto sigmoid, ascending and transverse colon each 20% respectively. 50% had stoma, 60% chemotherapy and 40% had radiotherapy. Karnofsky performance status was ≥80 in 50% of patients.

### Main Findings

There was no difficulty in understanding the introduction as well as the instructions for completion of the questionnaire. Mean duration for completion of a set was found to be 8 ± 2 minutes.

Patients consider the time of administration which was immediately after the consultation with the doctors to be inappropriate. Questionnaire items 1–28, 31–54 were not associated with any difficulty in answering, nor were they confusing, difficult to understand or offensive. Questions 29–30 and 56–59 were associated with some problems. Questions 29 &30 are questions about the overall quality of life and general health status**.** Three patients answering Bahasa-Malaysia versions indicated their difficulty in differentiating between question 29 & 30. They considered the duo to mean the same and suggested the questions to be merged. Questions 56 to 59 are questions about the sexual activities. (Questions 56–57 are for male and 58–59 are for female patients respectively). Three patients (Bahasa-Malaysia version) and five patients (Chinese-Malaysia version) felt the questions were not necessary because they were no more sexually active.

## Discussion

The pilot testing showed that the use of the Malaysian translated versions of EORTC QLQ C30, CR29 instruments is feasible in our sample of colorectal cancer patients. Instructions for completion as well as questions were well understood except the questions on the overall quality of life, overall health status and sexual activity. The use of self-administered interviewer delivered method of data collection was a good way to address this problem. Timing of administration was of major concern. We therefore, opted for another approach which was to ask the patients to complete the questionnaire while waiting for their turn to see doctor(s) as opposed to the initial method of administering the questionnaire after the consultation.

This study is the first of its kind among colorectal cancer patients in Malaysia. Previous studies in China, Taiwan and Singapore indicated good psychometric properties for the standard Chinese versions and English versions of the EORTC QLQ C-30 AND CR38 instruments respectively. [16-20]. The current version of the EORTC QLQ-CR29 underwent a number of validation studies since it was first developed and validated in Europe in 2009[9]. Such validation studies indicate an excellent psychometric property [21-23]. Interestingly, there was no such validation in Malaysia or its neighbors such as Singapore, Thailand or Brunei which might render this study unnecessary. We acknowledge one previous study involving the validation of Malay version of EORTC QLQ-C30 among breast cancer patients and a subsequent study using such instrument validated among breast cancer patients to study the quality of life of colorectal cancer patients.

Our initial plan was to study the psychometric properties of the instruments among the three commonly spoken languages in Malaysia that are Bahasa Malaysia, Chinese-Malaysia and Tamil-Malaysia. However, findings from the pilot testing indicated that there was inadequate number of patients that were able to read and write in the Tamil language. This might be due to the fact that colorectal cancer is not as common among the Indian patients as compared to Chinese and Malay patients. In addition, most of the Malaysian Indian population does not receive their education from Tamil-medium school. In view of this, we had to give up on the Tamil questionnaires in this study.

The number of colorectal cancer patients with liver metastasis was not many. Out of these patients, quite a significant number of them were not operable; hence shorten their survival Thus we decided to drop these QLQ LMC21 questionnaires in this study.

Further studies are therefore recommended for the validation of the liver metastasis through a prospective/longitudinal study involving a large number of hospitals across Malaysia. Also we recommend the use of the instruments (if found to be valid and reliable) for studies in the feasibility of its application in day to day clinical follow up, also to generate a reference values for Malaysian colorectal cancer patients that will be useful in future policy and research purposes.

In conclusion, it is clear that the use of the Malaysian versions of EORTC QLQ C30, CR29 instruments is feasible in our sample of colorectal cancer patients. Instructions for completion as well as questions were well understood except the questions on the overall quality of life, overall health status and sexual activity. We anticipate that once the validation is completed, it would provide adequate information on the psychometric properties of the Malaysian versions of the EORTC QLQ-C30 and CR29 for Bahasa Malaysia and Chinese Malaysia. In future, this validated instrument would be very useful in complementing the routine clinical practice involving colorectal cancer patients.

## Competing interests

The authors declare that they have no competing interest.

## Authors' contributions

BAM, FMM, CWL, ACR and JMB participated in the design and coordination of the study. FMM, BAM provides the statistical support. BAM, FMM, CWL, ACR, JMB, IS and JZ all reviewed the study protocol and made suggestions that improve the design. All of these individuals are involved in the management of the study. All of the authors read, revised and approved the final manuscript.

## Pre-publication history

The pre-publication history for this paper can be accessed here:

http://www.biomedcentral.com/1471-2407/12/384/prepub
